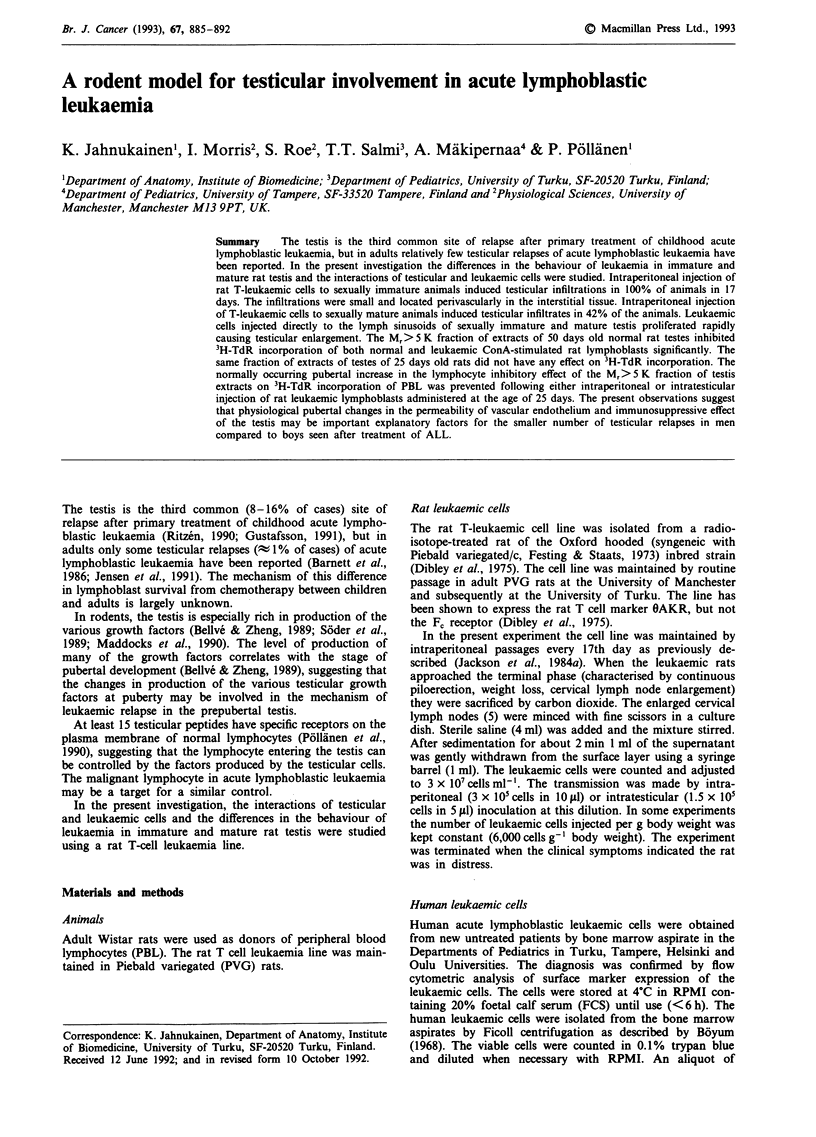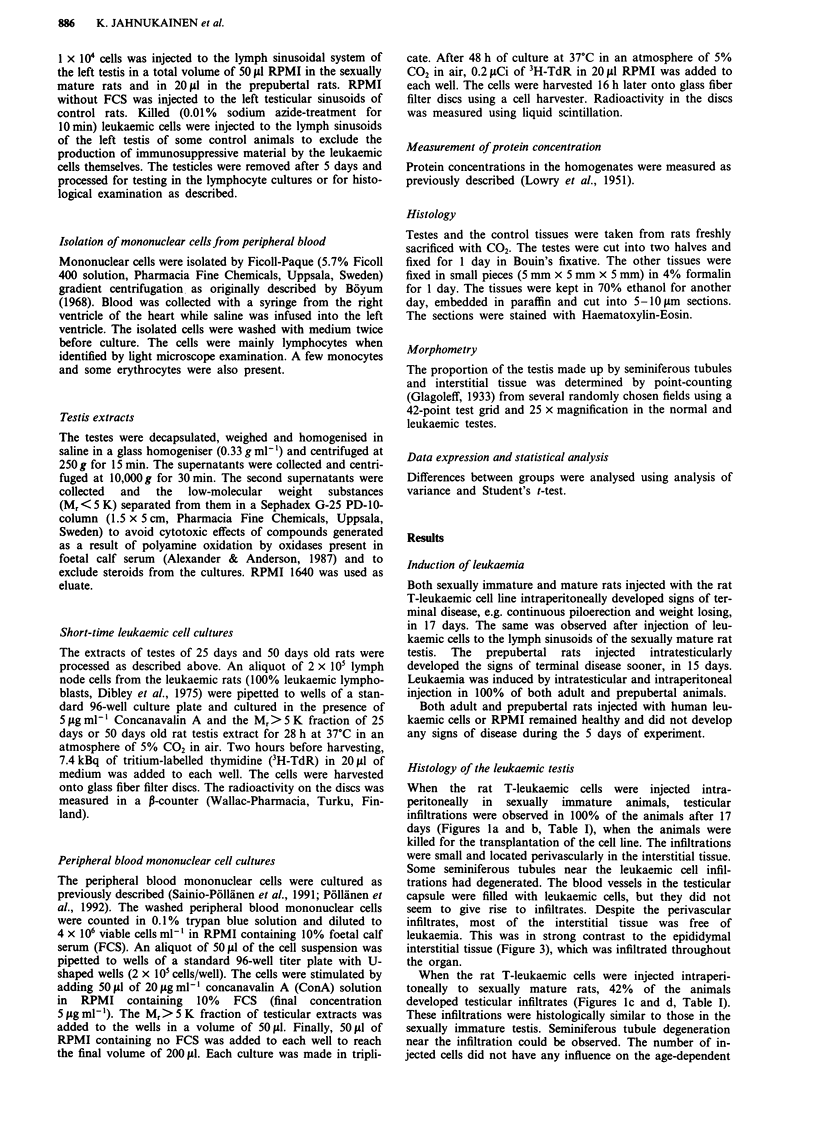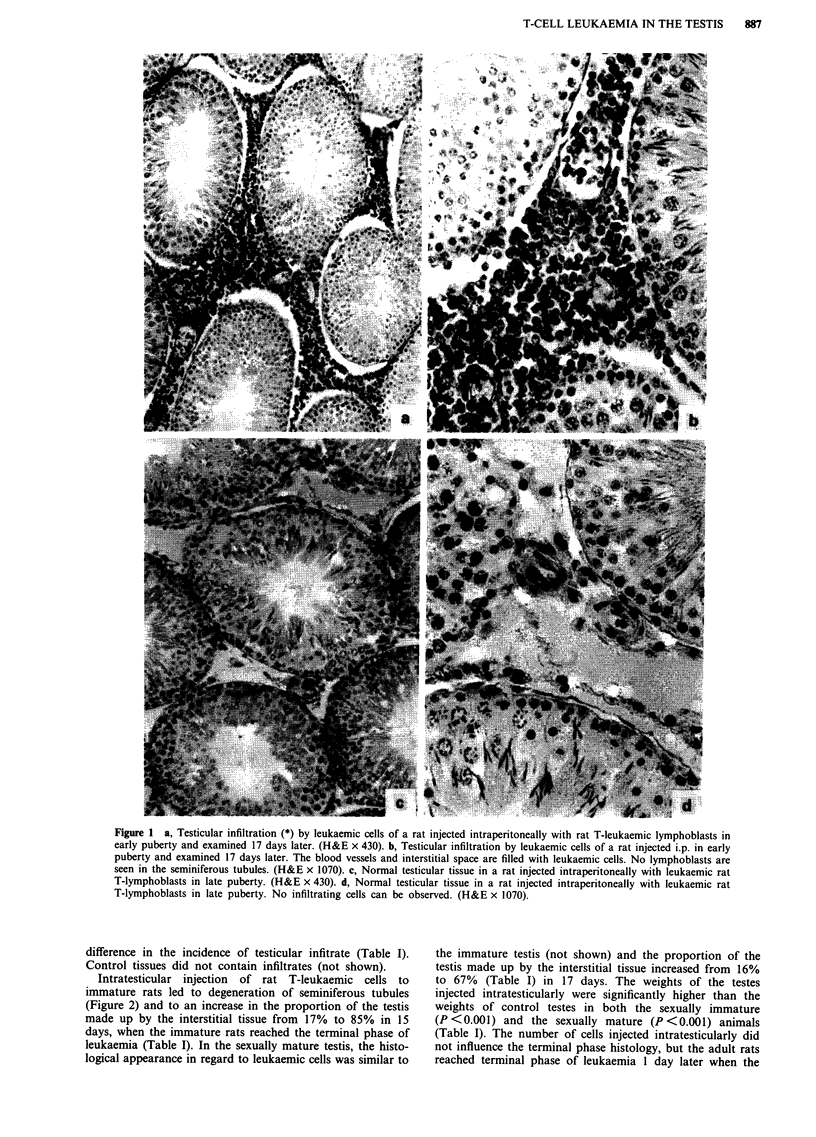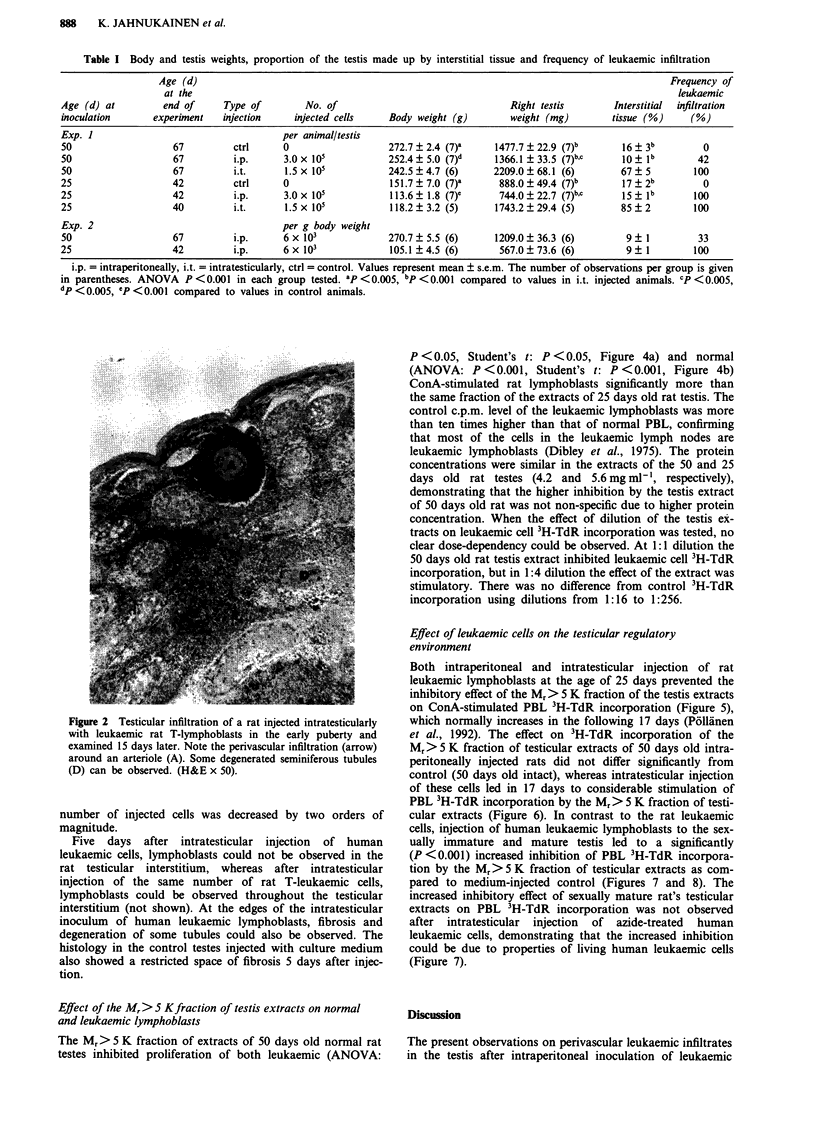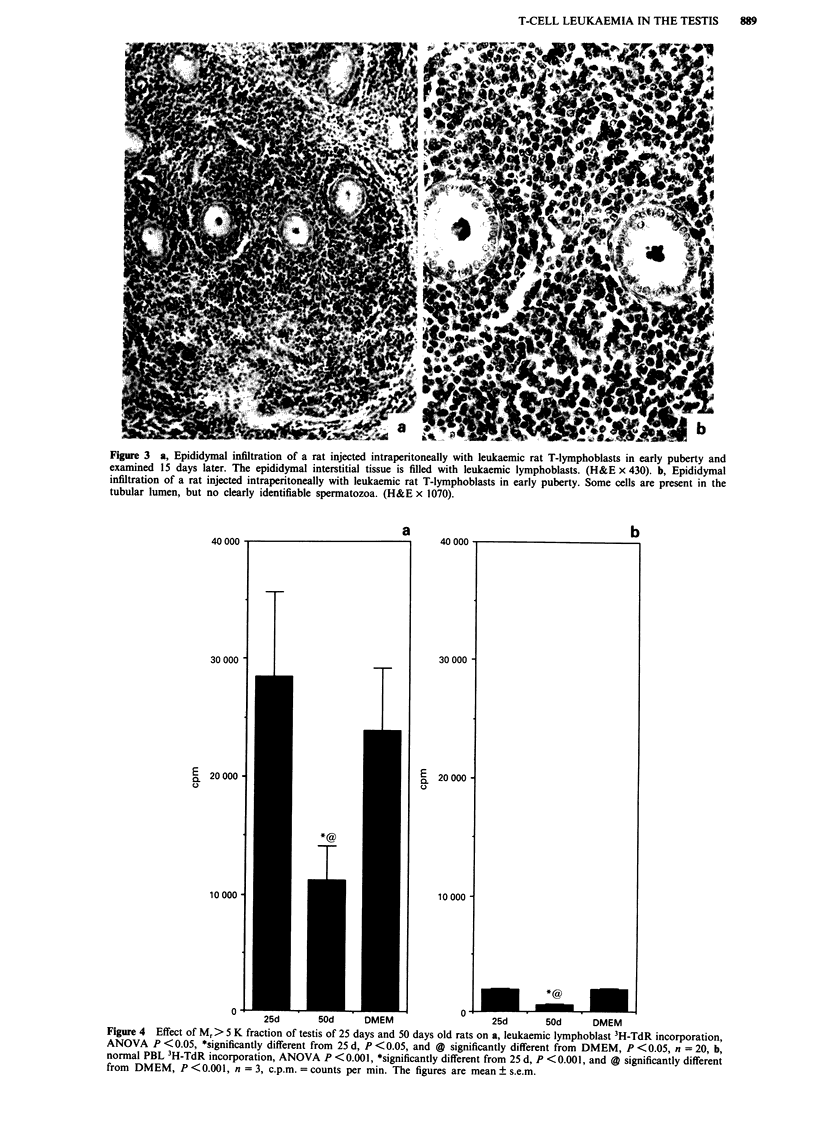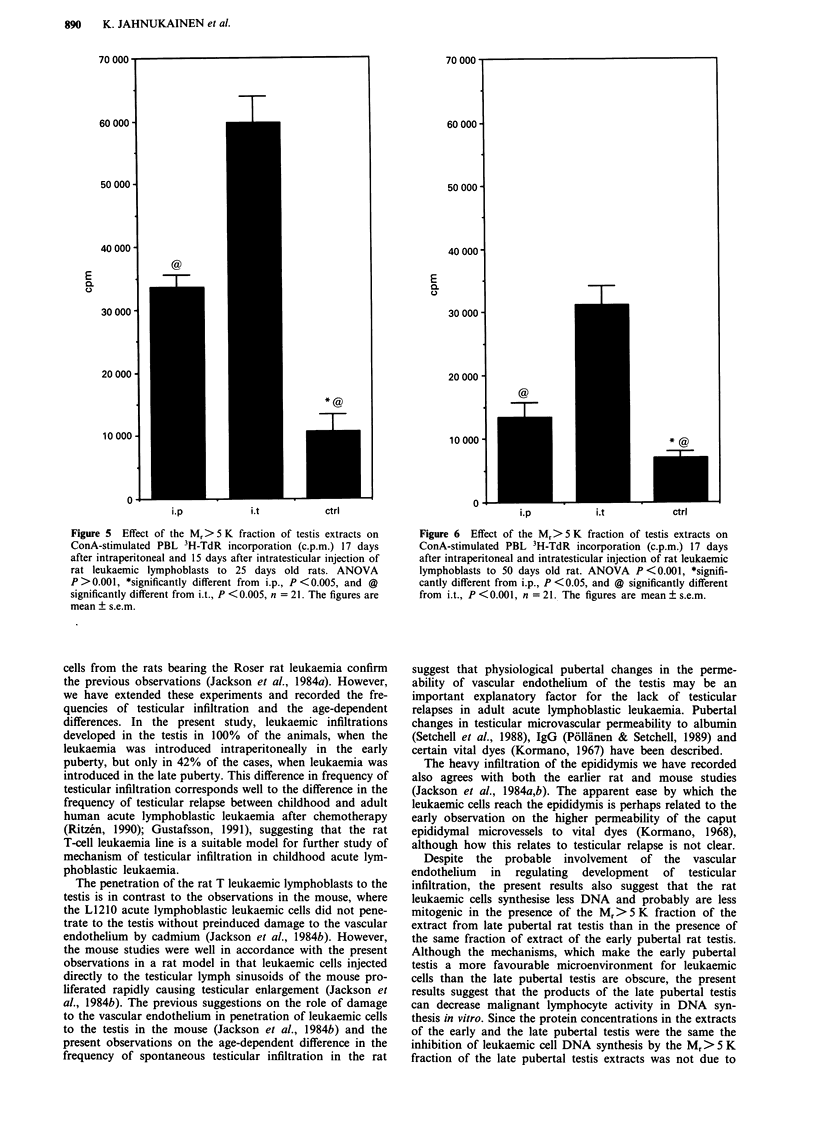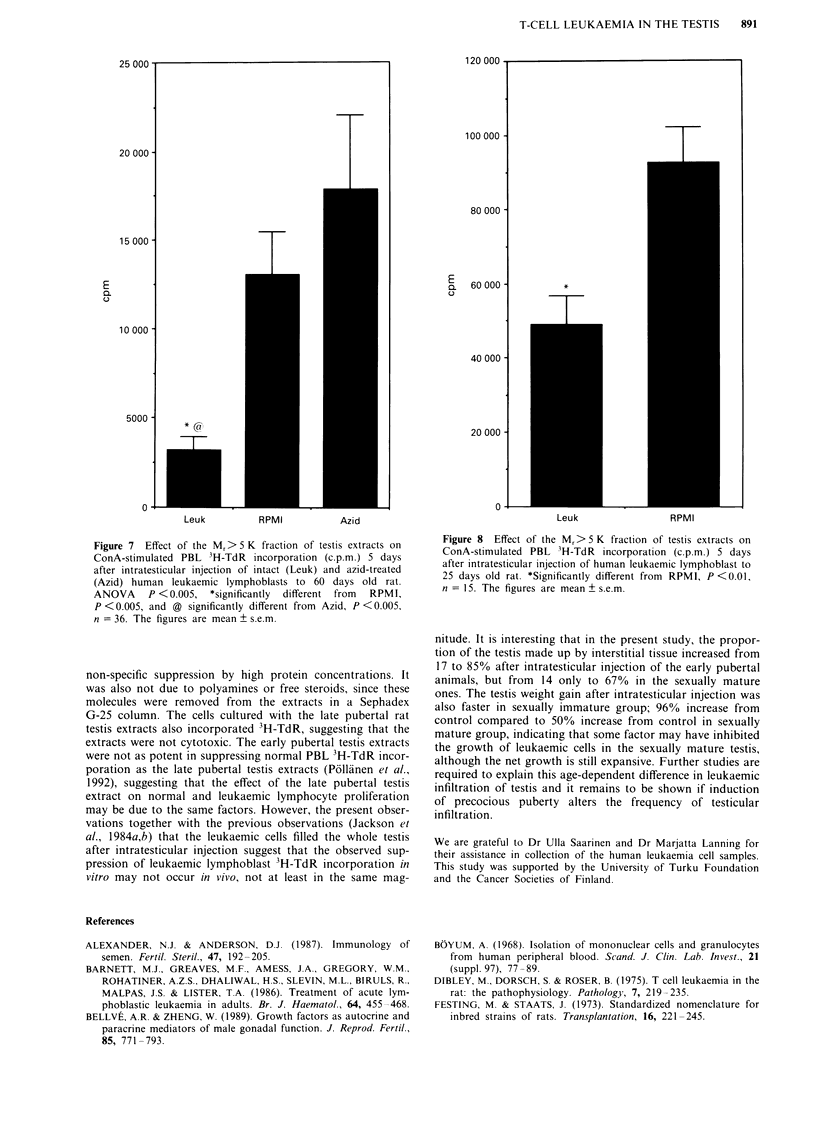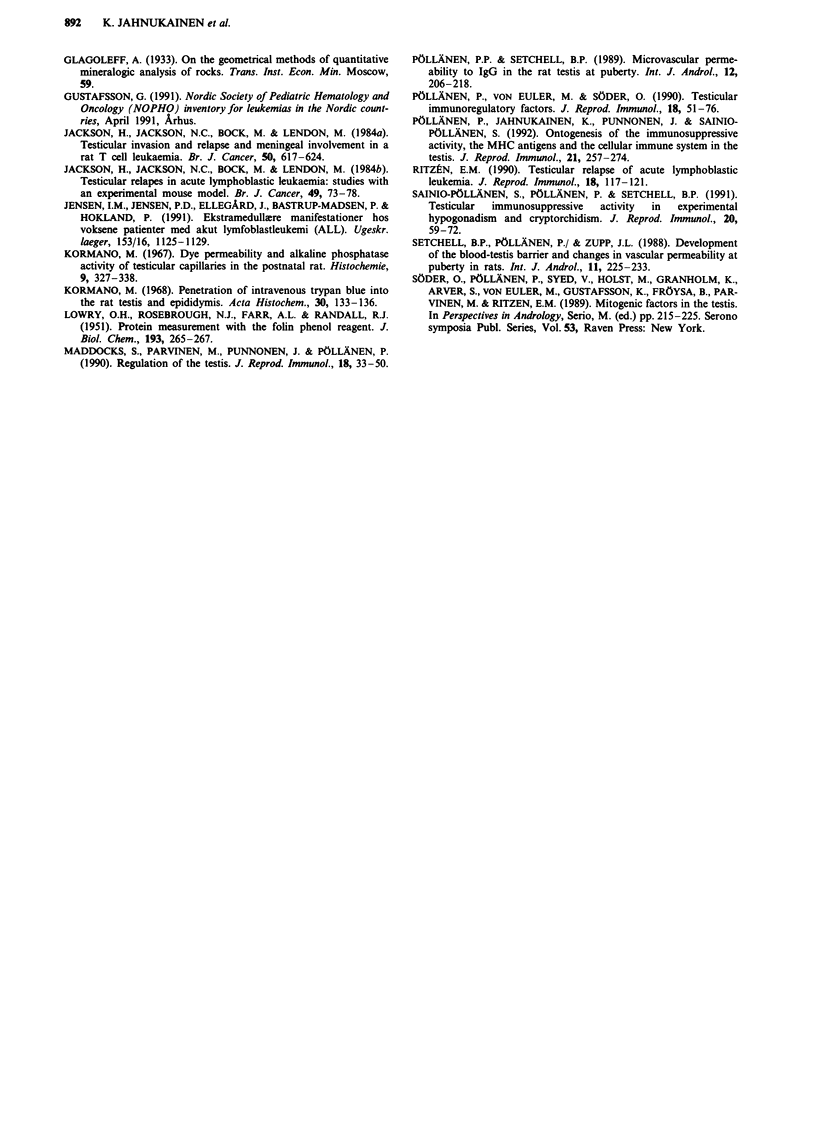# A rodent model for testicular involvement in acute lymphoblastic leukaemia.

**DOI:** 10.1038/bjc.1993.166

**Published:** 1993-05

**Authors:** K. Jahnukainen, I. Morris, S. Roe, T. T. Salmi, A. Mäkipernaa, P. Pöllänen

**Affiliations:** Department of Anatomy, University of Turku, Finland.

## Abstract

**Images:**


Br. J. Cancer (1993), 67, 885-892 © Macmillan Press Ltd., 1993

### A rodent model for testicular involvement in acute lymphoblastic leukaemia

K. Jahnukainen[1], I. Morris[2], S. Roe[2], T.T. Salmi[3], A. Mäkipernaa[4] & P. Pöllänen[1]

[1]*Department of Anatomy, Institute of Biomedicine;* [3]*Department of Pediatrics, University of Turku, SF-20520 Turku, Finland;* [4]*Department of Pediatrics, University of Tampere, SF-33520 Tampere, Finland and* [2]*Physiological Sciences, University of Manchester, Manchester M13 9PT, UK.*

**Summary** The testis is the third common site of relapse after primary treatment of childhood acute lymphoblastic leukaemia, but in adults relatively few testicular relapses of acute lymphoblastic leukaemia have been reported. In the present investigation the differences in the behaviour of leukaemia in immature and mature rat testis and the interactions of testicular and leukaemic cells were studied. Intraperitoneal injection of rat T-leukaemic cells to sexually immature animals induced testicular infiltrations in 100% of animals in 17 days. The infiltrations were small and located perivascularly in the interstitial tissue. Intraperitoneal injection of T-leukaemic cells to sexually mature animals induced testicular infiltrates in 42% of the animals. Leukaemic cells injected directly to the lymph sinusoids of sexually immature and mature testis proliferated rapidly causing testicular enlargement. The $M_r > 5$ K fraction of extracts of 50 days old normal rat testes inhibited $^3$H-TdR incorporation of both normal and leukaemic ConA-stimulated rat lymphoblasts significantly. The same fraction of extracts of testes of 25 days old rats did not have any effect on $^3$H-TdR incorporation. The normally occurring pubertal increase in the lymphocyte inhibitory effect of the $M_r > 5$ K fraction of testis extracts on $^3$H-TdR incorporation of PBL was prevented following either intraperitoneal or intratesticular injection of rat leukaemic lymphoblasts administered at the age of 25 days. The present observations suggest that physiological pubertal changes in the permeability of vascular endothelium and immunosuppressive effect of the testis may be important explanatory factors for the smaller number of testicular relapses in men compared to boys seen after treatment of ALL.

The testis is the third common (8–16% of cases) site of relapse after primary treatment of childhood acute lymphoblastic leukaemia (Ritzén, 1990; Gustafsson, 1991), but in adults only some testicular relapses (≈1% of cases) of acute lymphoblastic leukaemia have been reported (Barnett *et al.*, 1986; Jensen *et al.*, 1991). The mechanism of this difference in lymphoblast survival from chemotherapy between children and adults is largely unknown.

In rodents, the testis is especially rich in production of the various growth factors (Bellvé & Zheng, 1989; Söder *et al.*, 1989; Maddocks *et al.*, 1990). The level of production of many of the growth factors correlates with the stage of pubertal development (Bellvé & Zheng, 1989), suggesting that the changes in production of the various testicular growth factors at puberty may be involved in the mechanism of leukaemic relapse in the prepubertal testis.

At least 15 testicular peptides have specific receptors on the plasma membrane of normal lymphocytes (Pöllänen *et al.*, 1990), suggesting that the lymphocyte entering the testis can be controlled by the factors produced by the testicular cells. The malignant lymphocyte in acute lymphoblastic leukaemia may be a target for a similar control.

In the present investigation, the interactions of testicular and leukaemic cells and the differences in the behaviour of leukaemia in immature and mature rat testis were studied using a rat T-cell leukaemia line.

### Materials and methods

### *Animals*

Adult Wistar rats were used as donors of peripheral blood lymphocytes (PBL). The rat T cell leukaemia line was maintained in Piebald variegated (PVG) rats.

Correspondence: K. Jahnukainen, Department of Anatomy, Institute of Biomedicine, University of Turku, SF-20520 Turku, Finland.
Received 12 June 1992; and in revised form 10 October 1992.

### *Rat leukaemic cells*

The rat T-leukaemic cell line was isolated from a radioisotope-treated rat of the Oxford hooded (syngeneic with Piebald variegated/c, Festing & Staats, 1973) inbred strain (Dibley *et al.*, 1975). The cell line was maintained by routine passage in adult PVG rats at the University of Manchester and subsequently at the University of Turku. The line has been shown to express the rat T cell marker θAKR, but not the $F_c$ receptor (Dibley *et al.*, 1975).

In the present experiment the cell line was maintained by intraperitoneal passages every 17th day as previously described (Jackson *et al.*, 1984*a*). When the leukaemic rats approached the terminal phase (characterised by continuous piloerection, weight loss, cervical lymph node enlargement) they were sacrificed by carbon dioxide. The enlarged cervical lymph nodes (5) were minced with fine scissors in a culture dish. Sterile saline (4 ml) was added and the mixture stirred. After sedimentation for about 2 min 1 ml of the supernatant was gently withdrawn from the surface layer using a syringe barrel (1 ml). The leukaemic cells were counted and adjusted to $3 \times 10^7$ cells ml$^{-1}$. The transmission was made by intraperitoneal ($3 \times 10^5$ cells in 10 μl) or intratesticular ($1.5 \times 10^5$ cells in 5 μl) inoculation at this dilution. In some experiments the number of leukaemic cells injected per g body weight was kept constant (6,000 cells g$^{-1}$ body weight). The experiment was terminated when the clinical symptoms indicated the rat was in distress.

### *Human leukaemic cells*

Human acute lymphoblastic leukaemic cells were obtained from new untreated patients by bone marrow aspirate in the Departments of Pediatrics in Turku, Tampere, Helsinki and Oulu Universities. The diagnosis was confirmed by flow cytometric analysis of surface marker expression of the leukaemic cells. The cells were stored at 4°C in RPMI containing 20% foetal calf serum (FCS) until use (<6 h). The human leukaemic cells were isolated from the bone marrow aspirates by Ficoll centrifugation as described by Böyum (1968). The viable cells were counted in 0.1% trypan blue and diluted when necessary with RPMI. An aliquot of

$1 \times 10^4$ cells was injected to the lymph sinusoidal system of the left testis in a total volume of 50 µl RPMI in the sexually mature rats and in 20 µl in the prepubertal rats. RPMI without FCS was injected to the left testicular sinusoids of control rats. Killed (0.01% sodium azide-treatment for 10 min) leukaemic cells were injected to the lymph sinusoids of the left testis of some control animals to exclude the production of immunosuppressive material by the leukaemic cells themselves. The testicles were removed after 5 days and processed for testing in the lymphocyte cultures or for histological examination as described.

*Isolation of mononuclear cells from peripheral blood*

Mononuclear cells were isolated by Ficoll-Paque (5.7% Ficoll 400 solution, Pharmacia Fine Chemicals, Uppsala, Sweden) gradient centrifugation as originally described by Böyum (1968). Blood was collected with a syringe from the right ventricle of the heart while saline was infused into the left ventricle. The isolated cells were washed with medium twice before culture. The cells were mainly lymphocytes when identified by light microscope examination. A few monocytes and some erythrocytes were also present.

*Testis extracts*

The testes were decapsulated, weighed and homogenised in saline in a glass homogeniser (0.33 $g\ ml^{-1}$) and centrifuged at 250 $g$ for 15 min. The supernatants were collected and centrifuged at 10,000 $g$ for 30 min. The second supernatants were collected and the low-molecular weight substances ($M_r < 5$ K) separated from them in a Sephadex G-25 PD-10-column (1.5 × 5 cm, Pharmacia Fine Chemicals, Uppsala, Sweden) to avoid cytotoxic effects of compounds generated as a result of polyamine oxidation by oxidases present in foetal calf serum (Alexander & Anderson, 1987) and to exclude steroids from the cultures. RPMI 1640 was used as eluate.

*Short-time leukaemic cell cultures*

The extracts of testes of 25 days and 50 days old rats were processed as described above. An aliquot of $2 \times 10^5$ lymph node cells from the leukaemic rats (100% leukaemic lymphoblasts, Dibley et al., 1975) were pipetted to wells of a standard 96-well culture plate and cultured in the presence of 5 µg $ml^{-1}$ Concanavalin A and the $M_r > 5$ K fraction of 25 days or 50 days old rat testis extract for 28 h at 37°C in an atmosphere of 5% $CO_2$ in air. Two hours before harvesting, 7.4 kBq of tritium-labelled thymidine ($^3$H-TdR) in 20 µl of medium was added to each well. The cells were harvested onto glass fiber filter discs. The radioactivity on the discs was measured in a β-counter (Wallac-Pharmacia, Turku, Finland).

*Peripheral blood mononuclear cell cultures*

The peripheral blood mononuclear cells were cultured as previously described (Sainio-Pöllänen et al., 1991; Pöllänen et al., 1992). The washed peripheral blood mononuclear cells were counted in 0.1% trypan blue solution and diluted to $4 \times 10^6$ viable cells $ml^{-1}$ in RPMI containing 10% foetal calf serum (FCS). An aliquot of 50 µl of the cell suspension was pipetted to wells of a standard 96-well titer plate with U-shaped wells ($2 \times 10^5$ cells/well). The cells were stimulated by adding 50 µl of 20 µg $ml^{-1}$ concanavalin A (ConA) solution in RPMI containing 10% FCS (final concentration 5 µg $ml^{-1}$). The $M_r > 5$ K fraction of testicular extracts was added to the wells in a volume of 50 µl. Finally, 50 µl of RPMI containing no FCS was added to each well to reach the final volume of 200 µl. Each culture was made in triplicate. After 48 h of culture at 37°C in an atmosphere of 5% $CO_2$ in air, 0.2 µCi of $^3$H-TdR in 20 µl RPMI was added to each well. The cells were harvested 16 h later onto glass fiber filter discs using a cell harvester. Radioactivity in the discs was measured using liquid scintillation.

*Measurement of protein concentration*

Protein concentrations in the homogenates were measured as previously described (Lowry et al., 1951).

*Histology*

Testes and the control tissues were taken from rats freshly sacrificed with $CO_2$. The testes were cut into two halves and fixed for 1 day in Bouin's fixative. The other tissues were fixed in small pieces (5 mm × 5 mm × 5 mm) in 4% formalin for 1 day. The tissues were kept in 70% ethanol for another day, embedded in paraffin and cut into 5–10 µm sections. The sections were stained with Haematoxylin-Eosin.

*Morphometry*

The proportion of the testis made up by seminiferous tubules and interstitial tissue was determined by point-counting (Glagoleff, 1933) from several randomly chosen fields using a 42-point test grid and 25 × magnification in the normal and leukaemic testes.

*Data expression and statistical analysis*

Differences between groups were analysed using analysis of variance and Student's $t$-test.

### Results

*Induction of leukaemia*

Both sexually immature and mature rats injected with the rat T-leukaemic cell line intraperitoneally developed signs of terminal disease, e.g. continuous piloerection and weight losing, in 17 days. The same was observed after injection of leukaemic cells to the lymph sinusoids of the sexually mature rat testis. The prepubertal rats injected intratesticularly developed the signs of terminal disease sooner, in 15 days. Leukaemia was induced by intratesticular and intraperitoneal injection in 100% of both adult and prepubertal animals.

Both adult and prepubertal rats injected with human leukaemic cells or RPMI remained healthy and did not develop any signs of disease during the 5 days of experiment.

*Histology of the leukaemic testis*

When the rat T-leukaemic cells were injected intraperitoneally in sexually immature animals, testicular infiltrations were observed in 100% of the animals after 17 days (Figures 1a and b, Table I), when the animals were killed for the transplantation of the cell line. The infiltrations were small and located perivascularly in the interstitial tissue. Some seminiferous tubules near the leukaemic cell infiltrations had degenerated. The blood vessels in the testicular capsule were filled with leukaemic cells, but they did not seem to give rise to infiltrates. Despite the perivascular infiltrates, most of the interstitial tissue was free of leukaemia. This was in strong contrast to the epididymal interstitial tissue (Figure 3), which was infiltrated throughout the organ.

When the rat T-leukaemic cells were injected intraperitoneally to sexually mature rats, 42% of the animals developed testicular infiltrates (Figures 1c and d, Table I). These infiltrations were histologically similar to those in the sexually immature testis. Seminiferous tubule degeneration near the infiltration could be observed. The number of injected cells did not have any influence on the age-dependent

**Figure 1** **a**, Testicular infiltration (*) by leukaemic cells of a rat injected intraperitoneally with rat T-leukaemic lymphoblasts in early puberty and examined 17 days later. (H&E × 430). **b**, Testicular infiltration by leukaemic cells of a rat injected i.p. in early puberty and examined 17 days later. The blood vessels and interstitial space are filled with leukaemic cells. No lymphoblasts are seen in the seminiferous tubules. (H&E × 1070). **c**, Normal testicular tissue in a rat injected intraperitoneally with leukaemic rat T-lymphoblasts in late puberty. (H&E × 430). **d**, Normal testicular tissue in a rat injected intraperitoneally with leukaemic rat T-lymphoblasts in late puberty. No infiltrating cells can be observed. (H&E × 1070).

difference in the incidence of testicular infitrate (Table I). Control tissues did not contain infiltrates (not shown).

Intratesticular injection of rat T-leukaemic cells to immature rats led to degeneration of seminiferous tubules (Figure 2) and to an increase in the proportion of the testis made up by the interstitial tissue from 17% to 85% in 15 days, when the immature rats reached the terminal phase of leukaemia (Table I). In the sexually mature testis, the histological appearance in regard to leukaemic cells was similar to the immature testis (not shown) and the proportion of the testis made up by the interstitial tissue increased from 16% to 67% (Table I) in 17 days. The weights of the testes injected intratesticularly were significantly higher than the weights of control testes in both the sexually immature ($P < 0.001$) and the sexually mature ($P < 0.001$) animals (Table I). The number of cells injected intratesticularly did not influence the terminal phase histology, but the adult rats reached terminal phase of leukaemia 1 day later when the

**Table I** Body and testis weights, proportion of the testis made up by interstitial tissue and frequency of leukaemic infiltration

| *Age (d) at inoculation* | *Age (d) at the end of experiment* | *Type of injection* | *No. of injected cells* | *Body weight (g)* | *Right testis weight (mg)* | *Interstitial tissue (%)* | *Frequency of leukaemic infiltration (%)* |
|---|---|---|---|---|---|---|---|
| *Exp. 1* | | | *per animal/testis* | | | | |
| 50 | 67 | ctrl | 0 | $272.7 \pm 2.4$ (7)$^a$ | $1477.7 \pm 22.9$ (7)$^b$ | $16 \pm 3^b$ | 0 |
| 50 | 67 | i.p. | $3.0 \times 10^5$ | $252.4 \pm 5.0$ (7)$^d$ | $1366.1 \pm 33.5$ (7)$^{b,c}$ | $10 \pm 1^b$ | 42 |
| 50 | 67 | i.t. | $1.5 \times 10^5$ | $242.5 \pm 4.7$ (6) | $2209.0 \pm 68.1$ (6) | $67 \pm 5$ | 100 |
| 25 | 42 | ctrl | 0 | $151.7 \pm 7.0$ (7)$^a$ | $888.0 \pm 49.4$ (7)$^b$ | $17 \pm 2^b$ | 0 |
| 25 | 42 | i.p. | $3.0 \times 10^5$ | $113.6 \pm 1.8$ (7)$^e$ | $744.0 \pm 22.7$ (7)$^{b,c}$ | $15 \pm 1^b$ | 100 |
| 25 | 40 | i.t. | $1.5 \times 10^5$ | $118.2 \pm 3.2$ (5) | $1743.2 \pm 29.4$ (5) | $85 \pm 2$ | 100 |
| *Exp. 2* | | | *per g body weight* | | | | |
| 50 | 67 | i.p. | $6 \times 10^3$ | $270.7 \pm 5.5$ (6) | $1209.0 \pm 36.3$ (6) | $9 \pm 1$ | 33 |
| 25 | 42 | i.p. | $6 \times 10^3$ | $105.1 \pm 4.5$ (6) | $567.0 \pm 73.6$ (6) | $9 \pm 1$ | 100 |

i.p. = intraperitoneally, i.t. = intratesticularly, ctrl = control. Values represent mean ± s.e.m. The number of observations per group is given in parentheses. ANOVA $P < 0.001$ in each group tested. $^aP < 0.005$, $^bP < 0.001$ compared to values in i.t. injected animals. $^cP < 0.005$, $^dP < 0.005$, $^eP < 0.001$ compared to values in control animals.

**Figure 2** Testicular infiltration of a rat injected intratesticularly with leukaemic rat T-lymphoblasts in the early puberty and examined 15 days later. Note the perivascular infiltration (arrow) around an arteriole (A). Some degenerated seminiferous tubules (D) can be observed. (H&E × 50).

number of injected cells was decreased by two orders of magnitude.

Five days after intratesticular injection of human leukaemic cells, lymphoblasts could not be observed in the rat testicular interstitium, whereas after intratesticular injection of the same number of rat T-leukaemic cells, lymphoblasts could be observed throughout the testicular interstitium (not shown). At the edges of the intratesticular inoculum of human leukaemic lymphoblasts, fibrosis and degeneration of some tubules could also be observed. The histology in the control testes injected with culture medium also showed a restricted space of fibrosis 5 days after injection.

*Effect of the $M_r > 5$ K fraction of testis extracts on normal and leukaemic lymphoblasts*

The $M_r > 5$ K fraction of extracts of 50 days old normal rat testes inhibited proliferation of both leukaemic (ANOVA: $P < 0.05$, Student's $t$: $P < 0.05$, Figure 4a) and normal (ANOVA: $P < 0.001$, Student's $t$: $P < 0.001$, Figure 4b) ConA-stimulated rat lymphoblasts significantly more than the same fraction of the extracts of 25 days old rat testis. The control c.p.m. level of the leukaemic lymphoblasts was more than ten times higher than that of normal PBL, confirming that most of the cells in the leukaemic lymph nodes are leukaemic lymphoblasts (Dibley *et al.*, 1975). The protein concentrations were similar in the extracts of the 50 and 25 days old rat testes (4.2 and 5.6 mg ml$^{-1}$, respectively), demonstrating that the higher inhibition by the testis extract of 50 days old rat was not non-specific due to higher protein concentration. When the effect of dilution of the testis extracts on leukaemic cell $^3$H-TdR incorporation was tested, no clear dose-dependency could be observed. At 1:1 dilution the 50 days old rat testis extract inhibited leukaemic cell $^3$H-TdR incorporation, but in 1:4 dilution the effect of the extract was stimulatory. There was no difference from control $^3$H-TdR incorporation using dilutions from 1:16 to 1:256.

*Effect of leukaemic cells on the testicular regulatory environment*

Both intraperitoneal and intratesticular injection of rat leukaemic lymphoblasts at the age of 25 days prevented the inhibitory effect of the $M_r > 5$ K fraction of the testis extracts on ConA-stimulated PBL $^3$H-TdR incorporation (Figure 5), which normally increases in the following 17 days (Pöllänen *et al.*, 1992). The effect on $^3$H-TdR incorporation of the $M_r > 5$ K fraction of testicular extracts of 50 days old intraperitoneally injected rats did not differ significantly from control (50 days old intact), whereas intratesticular injection of these cells led in 17 days to considerable stimulation of PBL $^3$H-TdR incorporation by the $M_r > 5$ K fraction of testicular extracts (Figure 6). In contrast to the rat leukaemic cells, injection of human leukaemic lymphoblasts to the sexually immature and mature testis led to a significantly ($P < 0.001$) increased inhibition of PBL $^3$H-TdR incorporation by the $M_r > 5$ K fraction of testicular extracts as compared to medium-injected control (Figures 7 and 8). The increased inhibitory effect of sexually mature rat's testicular extracts on PBL $^3$H-TdR incorporation was not observed after intratesticular injection of azide-treated human leukaemic cells, demonstrating that the increased inhibition could be due to properties of living human leukaemic cells (Figure 7).

### Discussion

The present observations on perivascular leukaemic infiltrates in the testis after intraperitoneal inoculation of leukaemic

**Figure 3** **a**, Epididymal infiltration of a rat injected intraperitoneally with leukaemic rat T-lymphoblasts in early puberty and examined 15 days later. The epididymal interstitial tissue is filled with leukaemic lymphoblasts. (H&E × 430). **b**, Epididymal infiltration of a rat injected intraperitoneally with leukaemic rat T-lymphoblasts in early puberty. Some cells are present in the tubular lumen, but no clearly identifiable spermatozoa. (H&E × 1070).

**Figure 4** Effect of $M_r > 5$ K fraction of testis of 25 days and 50 days old rats on **a**, leukaemic lymphoblast $^3$H-TdR incorporation, ANOVA $P < 0.05$, *significantly different from 25 d, $P < 0.05$, and @ significantly different from DMEM, $P < 0.05$, $n = 20$, **b**, normal PBL $^3$H-TdR incorporation, ANOVA $P < 0.001$, *significantly different from 25 d, $P < 0.001$, and @ significantly different from DMEM, $P < 0.001$, $n = 3$, c.p.m. = counts per min. The figures are mean ± s.e.m.

**Figure 5** Effect of the $M_r > 5$ K fraction of testis extracts on ConA-stimulated PBL $^3$H-TdR incorporation (c.p.m.) 17 days after intraperitoneal and 15 days after intratesticular injection of rat leukaemic lymphoblasts to 25 days old rats. ANOVA $P > 0.001$, *significantly different from i.p., $P < 0.005$, and @ significantly different from i.t., $P < 0.005$, $n = 21$. The figures are mean ± s.e.m.

**Figure 6** Effect of the $M_r > 5$ K fraction of testis extracts on ConA-stimulated PBL $^3$H-TdR incorporation (c.p.m.) 17 days after intraperitoneal and intratesticular injection of rat leukaemic lymphoblasts to 50 days old rat. ANOVA $P < 0.001$, *significantly different from i.p., $P < 0.05$, and @ significantly different from i.t., $P < 0.001$, $n = 21$. The figures are mean ± s.e.m.

cells from the rats bearing the Roser rat leukaemia confirm the previous observations (Jackson *et al.*, 1984*a*). However, we have extended these experiments and recorded the frequencies of testicular infiltration and the age-dependent differences. In the present study, leukaemic infiltrations developed in the testis in 100% of the animals, when the leukaemia was introduced intraperitoneally in the early puberty, but only in 42% of the cases, when leukaemia was introduced in the late puberty. This difference in frequency of testicular infiltration corresponds well to the difference in the frequency of testicular relapse between childhood and adult human acute lymphoblastic leukaemia after chemotherapy (Ritzén, 1990); Gustafsson, 1991), suggesting that the rat T-cell leukaemia line is a suitable model for further study of mechanism of testicular infiltration in childhood acute lymphoblastic leukaemia.

The penetration of the rat T leukaemic lymphoblasts to the testis is in contrast to the observations in the mouse, where the L1210 acute lymphoblastic leukaemic cells did not penetrate to the testis without preinduced damage to the vascular endothelium by cadmium (Jackson *et al.*, 1984*b*). However, the mouse studies were well in accordance with the present observations in a rat model in that leukaemic cells injected directly to the testicular lymph sinusoids of the mouse proliferated rapidly causing testicular enlargement (Jackson *et al.*, 1984*b*). The previous suggestions on the role of damage to the vascular endothelium in penetration of leukaemic cells to the testis in the mouse (Jackson *et al.*, 1984*b*) and the present observations on the age-dependent difference in the frequency of spontaneous testicular infiltration in the rat suggest that physiological pubertal changes in the permeability of vascular endothelium of the testis may be an important explanatory factor for the lack of testicular relapses in adult acute lymphoblastic leukaemia. Pubertal changes in testicular microvascular permeability to albumin (Setchell *et al.*, 1988), IgG (Pöllänen & Setchell, 1989) and certain vital dyes (Kormano, 1967) have been described.

The heavy infiltration of the epididymis we have recorded also agrees with both the earlier rat and mouse studies (Jackson *et al.*, 1984*a*,*b*). The apparent ease by which the leukaemic cells reach the epididymis is perhaps related to the early observation on the higher permeability of the caput epididymal microvessels to vital dyes (Kormano, 1968), although how this relates to testicular relapse is not clear.

Despite the probable involvement of the vascular endothelium in regulating development of testicular infiltration, the present results also suggest that the rat leukaemic cells synthesise less DNA and probably are less mitogenic in the presence of the $M_r > 5$ K fraction of the extract from late pubertal rat testis than in the presence of the same fraction of extract of the early pubertal rat testis. Although the mechanisms, which make the early pubertal testis a more favourable microenvironment for leukaemic cells than the late pubertal testis are obscure, the present results suggest that the products of the late pubertal testis can decrease malignant lymphocyte activity in DNA synthesis *in vitro*. Since the protein concentrations in the extracts of the early and the late pubertal testis were the same the inhibition of leukaemic cell DNA synthesis by the $M_r > 5$ K fraction of the late pubertal testis extracts was not due to

**Figure 7** Effect of the $M_r > 5$ K fraction of testis extracts on ConA-stimulated PBL $^3$H-TdR incorporation (c.p.m.) 5 days after intratesticular injection of intact (Leuk) and azid-treated (Azid) human leukaemic lymphoblasts to 60 days old rat. ANOVA $P < 0.005$, *significantly different from RPMI, $P < 0.005$, and @ significantly different from Azid, $P < 0.005$, $n = 36$. The figures are mean ± s.e.m.

**Figure 8** Effect of the $M_r > 5$ K fraction of testis extracts on ConA-stimulated PBL $^3$H-TdR incorporation (c.p.m.) 5 days after intratesticular injection of human leukaemic lymphoblast to 25 days old rat. *Significantly different from RPMI, $P < 0.01$, $n = 15$. The figures are mean ± s.e.m.

non-specific suppression by high protein concentrations. It was also not due to polyamines or free steroids, since these molecules were removed from the extracts in a Sephadex G-25 column. The cells cultured with the late pubertal rat testis extracts also incorporated $^3$H-TdR, suggesting that the extracts were not cytotoxic. The early pubertal testis extracts were not as potent in suppressing normal PBL $^3$H-TdR incorporation as the late pubertal testis extracts (Pöllänen *et al.*, 1992), suggesting that the effect of the late pubertal testis extract on normal and leukaemic lymphocyte proliferation may be due to the same factors. However, the present observations together with the previous observations (Jackson *et al.*, 1984*a*,*b*) that the leukaemic cells filled the whole testis after intratesticular injection suggest that the observed suppression of leukaemic lymphoblast $^3$H-TdR incorporation *in vitro* may not occur *in vivo*, not at least in the same magnitude. It is interesting that in the present study, the proportion of the testis made up by interstitial tissue increased from 17 to 85% after intratesticular injection of the early pubertal animals, but from 14 only to 67% in the sexually mature ones. The testis weight gain after intratesticular injection was also faster in sexually immature group; 96% increase from control compared to 50% increase from control in sexually mature group, indicating that some factor may have inhibited the growth of leukaemic cells in the sexually mature testis, although the net growth is still expansive. Further studies are required to explain this age-dependent difference in leukaemic infiltration of testis and it remains to be shown if induction of precocious puberty alters the frequency of testicular infiltration.

We are grateful to Dr Ulla Saarinen and Dr Marjatta Lanning for their assistance in collection of the human leukaemia cell samples. This study was supported by the University of Turku Foundation and the Cancer Societies of Finland.

### References

ALEXANDER, N.J. & ANDERSON, D.J. (1987). Immunology of semen. *Fertil. Steril.*, **47**, 192–205.

BARNETT, M.J., GREAVES, M.F., AMESS, J.A., GREGORY, W.M., ROHATINER, A.Z.S., DHALIWAL, H.S., SLEVIN, M.L., BIRULS, R., MALPAS, J.S. & LISTER, T.A. (1986). Treatment of acute lymphoblastic leukaemia in adults. *Br. J. Haematol.*, **64**, 455–468.

BELLVÉ, A.R. & ZHENG, W. (1989). Growth factors as autocrine and paracrine mediators of male gonadal function. *J. Reprod. Fertil.*, **85**, 771–793.

BÖYUM, A. (1968). Isolation of mononuclear cells and granulocytes from human peripheral blood. *Scand. J. Clin. Lab. Invest.*, **21** (suppl. 97), 77–89.

DIBLEY, M., DORSCH, S. & ROSER, B. (1975). T cell leukaemia in the rat: the pathophysiology. *Pathology*, **7**, 219–235.

FESTING, M. & STAATS, J. (1973). Standardized nomenclature for inbred strains of rats. *Transplantation*, **16**, 221–245.

GLAGOLEFF, A. (1933). On the geometrical methods of quantitative mineralogic analysis of rocks. *Trans. Inst. Econ. Min.* Moscow, **59**.

GUSTAFSSON, G. (1991). *Nordic Society of Pediatric Hematology and Oncology (NOPHO) inventory for leukemias in the Nordic countries*, April 1991, Århus.

JACKSON, H., JACKSON, N.C., BOCK, M. & LENDON, M. (1984*a*). Testicular invasion and relapse and meningeal involvement in a rat T cell leukaemia. *Br. J. Cancer*, **50**, 617–624.

JACKSON, H., JACKSON, N.C., BOCK, M. & LENDON, M. (1984*b*). Testicular relapes in acute lymphoblastic leukaemia: studies with an experimental mouse model. *Br. J. Cancer*, **49**, 73–78.

JENSEN, I.M., JENSEN, P.D., ELLEGÅRD, J., BASTRUP-MADSEN, P. & HOKLAND, P. (1991). Ekstramedullære manifestationer hos voksene patienter med akut lymfoblastleukemi (ALL). *Ugeskr. laeger*, 153/16, 1125–1129.

KORMANO, M. (1967). Dye permeability and alkaline phosphatase activity of testicular capillaries in the postnatal rat. *Histochemie*, **9**, 327–338.

KORMANO, M. (1968). Penetration of intravenous trypan blue into the rat testis and epididymis. *Acta Histochem.*, **30**, 133–136.

LOWRY, O.H., ROSEBROUGH, N.J., FARR, A.L. & RANDALL, R.J. (1951). Protein measurement with the folin phenol reagent. *J. Biol. Chem.*, **193**, 265–267.

MADDOCKS, S., PARVINEN, M., PUNNONEN, J. & PÖLLÄNEN, P. (1990). Regulation of the testis. *J. Reprod. Immunol.*, **18**, 33–50.

PÖLLÄNEN, P.P. & SETCHELL, B.P. (1989). Microvascular permeability to IgG in the rat testis at puberty. *Int. J. Androl.*, **12**, 206–218.

PÖLLÄNEN, P., VON EULER, M. & SÖDER, O. (1990). Testicular immunoregulatory factors. *J. Reprod. Immunol.*, **18**, 51–76.

PÖLLÄNEN, P., JAHNUKAINEN, K., PUNNONEN, J. & SAINIO-PÖLLÄNEN, S. (1992). Ontogenesis of the immunosuppressive activity, the MHC antigens and the cellular immune system in the testis. *J. Reprod. Immunol.*, **21**, 257–274.

RITZÉN, E.M. (1990). Testicular relapse of acute lymphoblastic leukemia. *J. Reprod. Immunol.*, **18**, 117–121.

SAINIO-PÖLLÄNEN, S., PÖLLÄNEN, P. & SETCHELL, B.P. (1991). Testicular immunosuppressive activity in experimental hypogonadism and cryptorchidism. *J. Reprod. Immunol.*, **20**, 59–72.

SETCHELL, B.P., PÖLLÄNEN, P./ & ZUPP, J.L. (1988). Development of the blood-testis barrier and changes in vascular permeability at puberty in rats. *Int. J. Androl.*, **11**, 225–233.

SÖDER, O., PÖLLÄNEN, P., SYED, V., HOLST, M., GRANHOLM, K., ARVER, S., VON EULER, M., GUSTAFSSON, K., FRÖYSA, B., PARVINEN, M. & RITZEN, E.M. (1989). Mitogenic factors in the testis. In *Perspectives in Andrology*, Serio, M. (ed.) pp. 215–225. Serono symposia Publ. Series, Vol. **53**, Raven Press: New York.